# Anger and aggression research: A bibliometric analysis from 2012 to 2022

**DOI:** 10.1097/MD.0000000000035132

**Published:** 2023-09-08

**Authors:** Xiaowen Sun, Xufeng Yu, Kejian Li

**Affiliations:** a College of Traditional Chinese Medicine, Shandong University of Traditional Chinese Medicine, Jinan, Shandong Province, China.

**Keywords:** aggression, anger, CiteSpace, scientometrics analysis, visualization

## Abstract

Anger and aggression are common sources of distress and impairment. There is, however, no available data on anger and aggression based on bibliometric analysis. This study uses bibliometric analysis to analyze research hotspots and trends in anger and aggression. Publications on anger and aggression within the last ten years were collected from the Web of Science Core Collection. Using descriptive bibliometrics, journals, countries, institutions, authors, references, and keywords in anger and aggression research were visually analyzed via CiteSpace. A total of 3114 articles were included, and studies on anger and aggression increased yearly. The publications are mainly from 106 countries led by the USA and 381 institutions led by Univ Penn. We identified 505 authors, where Emil F. Coccaro had the highest number of articles, while Buss A.H. was the most frequently co-cited author. AGGRESSIVE BEHAVIOR is the journal that bore most of the studies, while PLOS ONE was the most cited journal. Our analysis demonstrated that research on anger and aggression is flourishing. Behaviors of anger and aggression, risk factors, neural mechanisms, personality, and adolescence have been researched hotspots in the past ten years. Besides, victimization, drosophila melanogaster, psychopathic traits, and perpetration are emerging anger and aggression research trends.

## 1. Introduction

Anger is a feeling of resentment or a desire for revenge, while aggression results from negative emotions, which is universal.^[1,2]^ It is reported that the prevalence rates for extreme anger and aggression in military personnel were 74% and 28%, respectively.^[3]^ On the other hand, most adolescents have a moderate level of anger, and 41% of the adolescents express their anger by shouting and fighting.^[4]^ Symptoms related to anger and aggression have been reported and include increased anger in premenstrual women,^[5,6]^ postpartum women,^[7]^ menopausal women,^[8]^ and other psychiatric outpatients.^[9–11]^ Anger and aggression are personality traits associated with suicide attempts, a major risk factor for suicide commission. Thus, as significant negative emotions, anger and aggression have elicited enormous research interests and are a heavy burden to the family and society, especially during the COVID-19 pandemic.^[12–14]^

Due to the effects of anger, mechanisms, and management of anger have become prevalent. However, data on the scientometric analysis of anger and aggression remains scant. Bibliometrics, first proposed in 1969, can qualitatively and quantitatively evaluate literature research trends,^[15]^ which not only help scholars quickly grasp research hotspots and development trends in specific research fields but also evaluate the distribution of countries/regions, authors, and journals in research fields, to lay a foundation for the development of future studies.^[16]^ CiteSpace is a unique tool in information visualization analysis,^[17]^ which has been applied in different fields to determine the status and trends of the research.^[18–20]^

This study explores the past ten years’ hotspots and development trends of anger and aggression. It draws a map of scientific knowledge via CiteSpace, to provide new insights for basic research and clinical prevention and treatment.

## 2. Materials and methods

### 2.1. Source database and retrieval strategy

The Web of Science Core Collection (WoSCC) was selected as the bibliometric analysis and data retrieval source database. The search strategy was as follows: TI = anger OR aggression between April 17, 2012 and April 17, 2022. The language was restricted to English, and only articles were included.

### 2.2. Data acquisition

**Figure 1.**
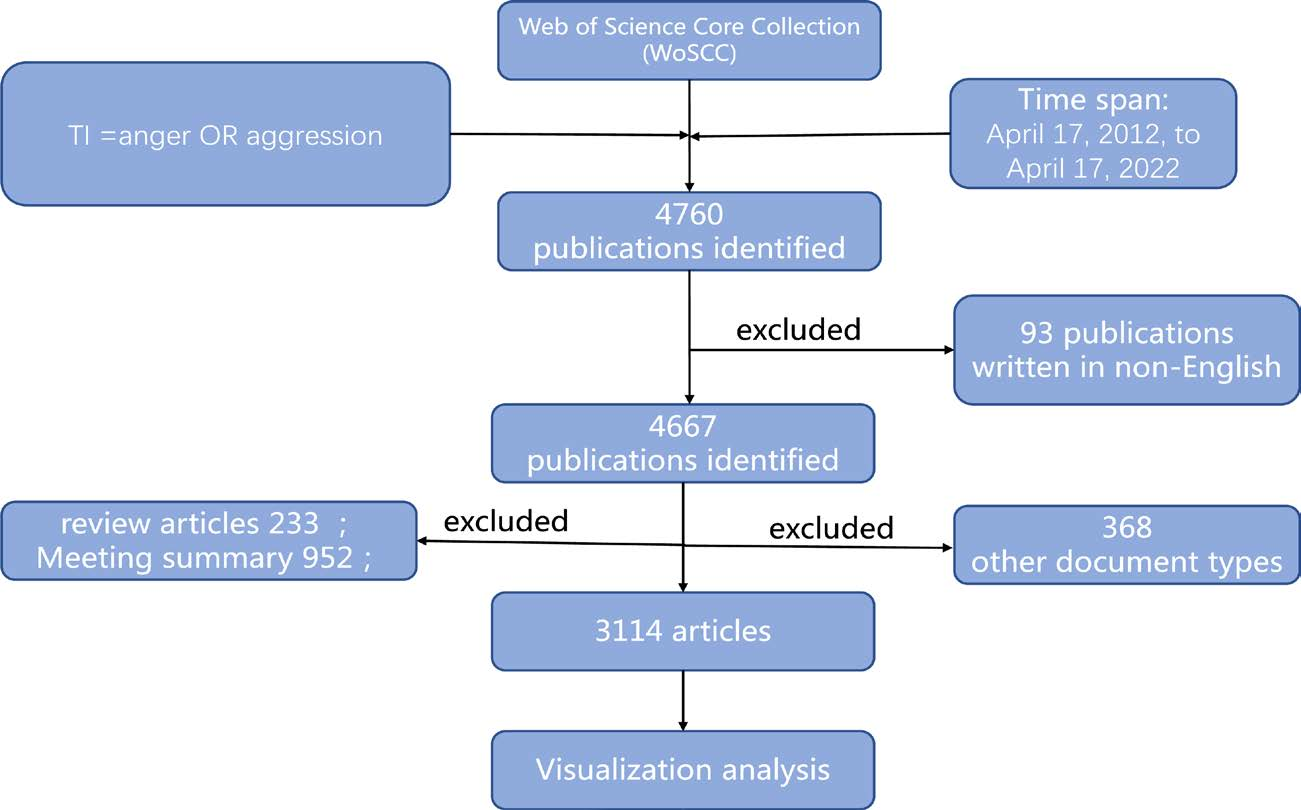
Flow diagram of processing publications.

Search results with “Full Record and Cited References” record content and a “Plain Text” file format were acquired. The files were renamed and then imported into CiteSpace for further analysis. The study flow diagram is shown in Figure 1. The ethical approval was unnecessary because the data do not contain any privacy information of patients.

### 2.3. Data analysis and visualization

We performed data analysis using Microsoft Office Excel 2021 and CiteSpace v5.8.R2. Microsoft Office Excel 2021 analyzed the annual publication output and trend. CiteSpace was used to produce visualized maps for categories, journals, countries, institutions, authors, co-cited references, keywords, and burst detection for global status and emerging trends in research on anger and aggression in the past 10 years.

## 3. Results

### 3.1. Trend of publication output

**Figure 2.**
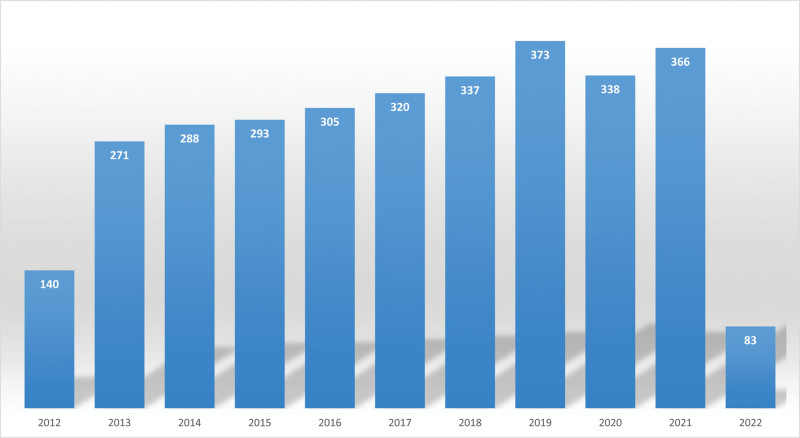
Publication outputs of anger and aggression research.

The number of publications published each year reflects the research development trend. As shown in Figure 2, a total of 3114 publications were retrieved. The number of publications increased from 140 in 2012 to 373 in 2019, demonstrating a stable upward trend. After 2019, there was a decline in literature output in this field, but it still remains high.

### 3.2. Contributing countries (regions) and institutions

**Figure 3.**
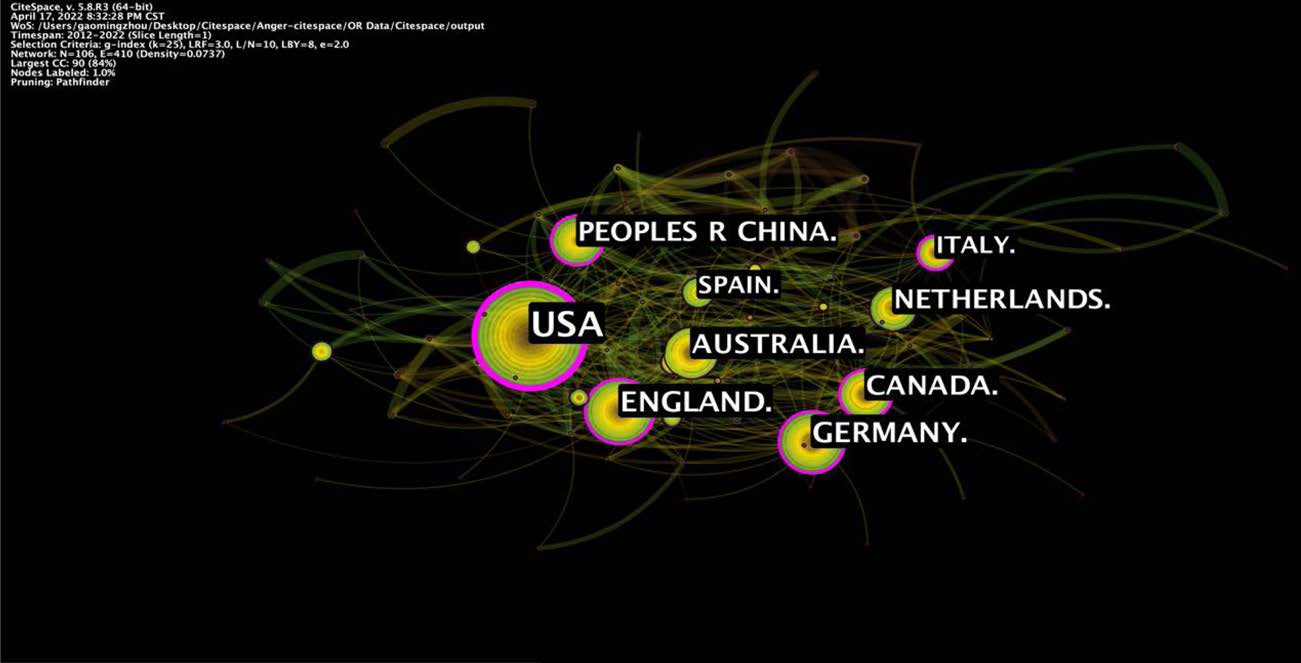
Map showing the distribution of countries.

**Figure 4.**
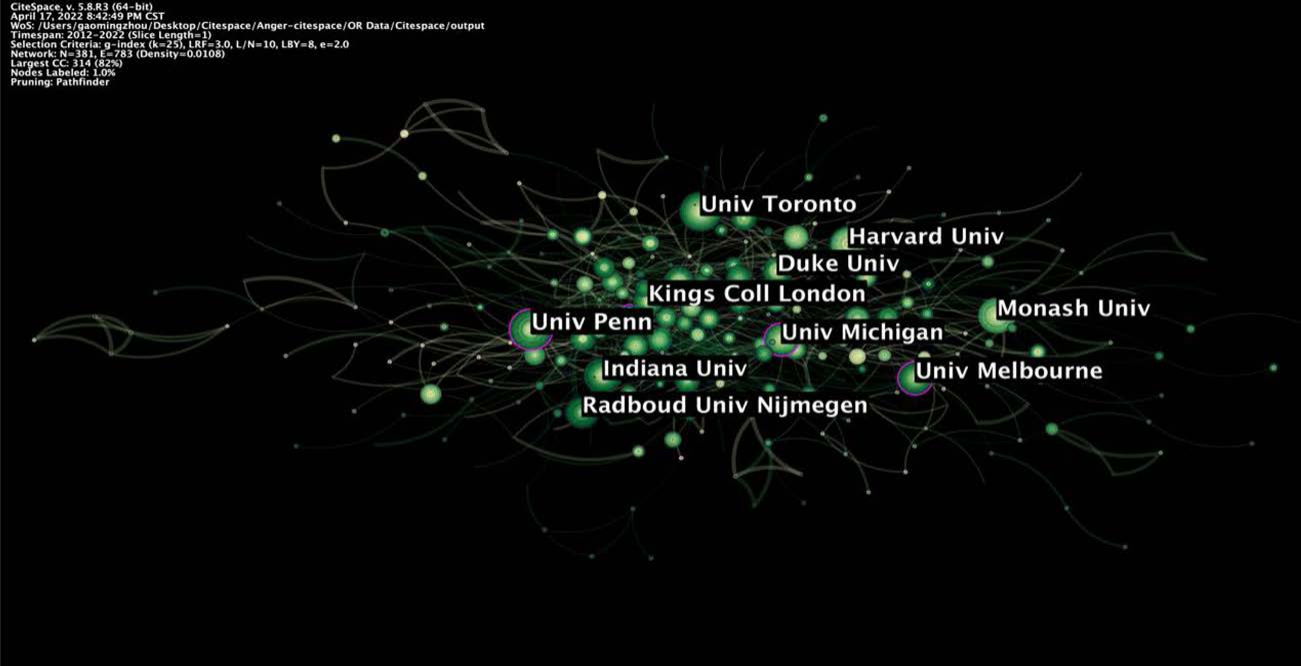
Map showing the distribution of institutions.

Our analysis showed that 3114 publications were published from 106 countries and 381 institutions. Among the contributing countries, the USA had the highest research output (1284 publications), with the highest centrality (0.26), demonstrating its leading role in the field. Although the number of papers published in China (212 publications, ranked 5th) was not the largest, it plays a major role in contributing to research in this field. Besides, analysis of publications in terms of centrality indicated that England and Germany also led the research development in this field (Fig. 3, Table 1). Of the 381 institutions, Univ Penn ranked first in the number of publications and centrality (Table 2). As shown in Figure 4, Univ Penn, Univ Melbourne, and Niv Michigan are leading institutions in centrality (Fig. 4).

**Table 1:** The top 10 prolific countries with the highest frequency and centrality of anger and aggression research in the past 10 yr.

Rank	Country	Publications	Country	Centrality
1	USA	1284	USA	0.26
2	England	285	Peoples R. China	0.16
3	Germany	263	Italy	0.15
4	Australia	242	Germany	0.14
5	Peoples R. China	212	England	0.11
6	Canada	205	Canada	0.11
7	Netherlands	191	Sweden	0.08
8	Spain	120	Spain	0.07
9	Italy	119	Australia	0.06
10	Switzerland	98	Switzerland	0.06

**Table 2:** Top 10 prolific institutions with the highest frequency and centrality of anger and aggression research in the past 10 yr.

Rank	Publications	Institution	Centrality	Institution
1	43	Univ Penn	0.12	Univ Penn
2	42	Harvard Univ	0.12	Univ Melbourne
3	42	Monash Univ	0.11	Univ Michigan
4	42	Univ Toronto	0.11	Emory Univ
5	40	Univ Melbourne	0.09	Columbia Univ
6	39	Radboud Univ Nijmegen	0.08	Radboud Univ Nijmegen
7	38	Duke Univ	0.07	Duke Univ
8	38	Kings Coll London	0.07	Boston Univ
9	35	Univ Michigan	0.07	Brown Univ
10	35	Indiana Univ	0.06	Harvard Univ

### 3.3. Analysis of authors and co-cited authors

**Figure 5.**
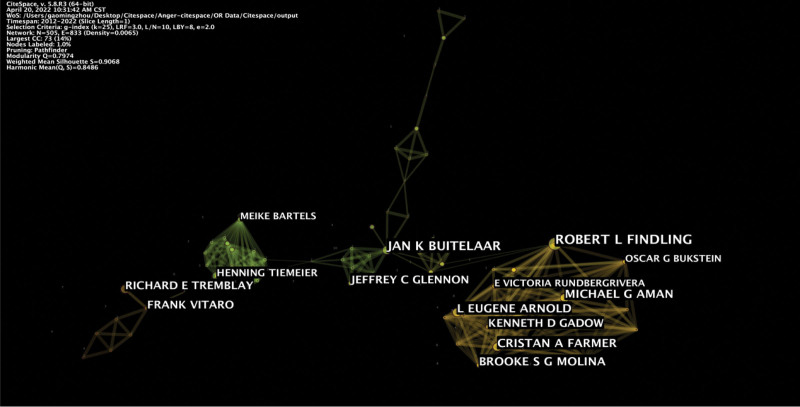
Map of authors involved in anger and aggression research.

**Figure 6.**
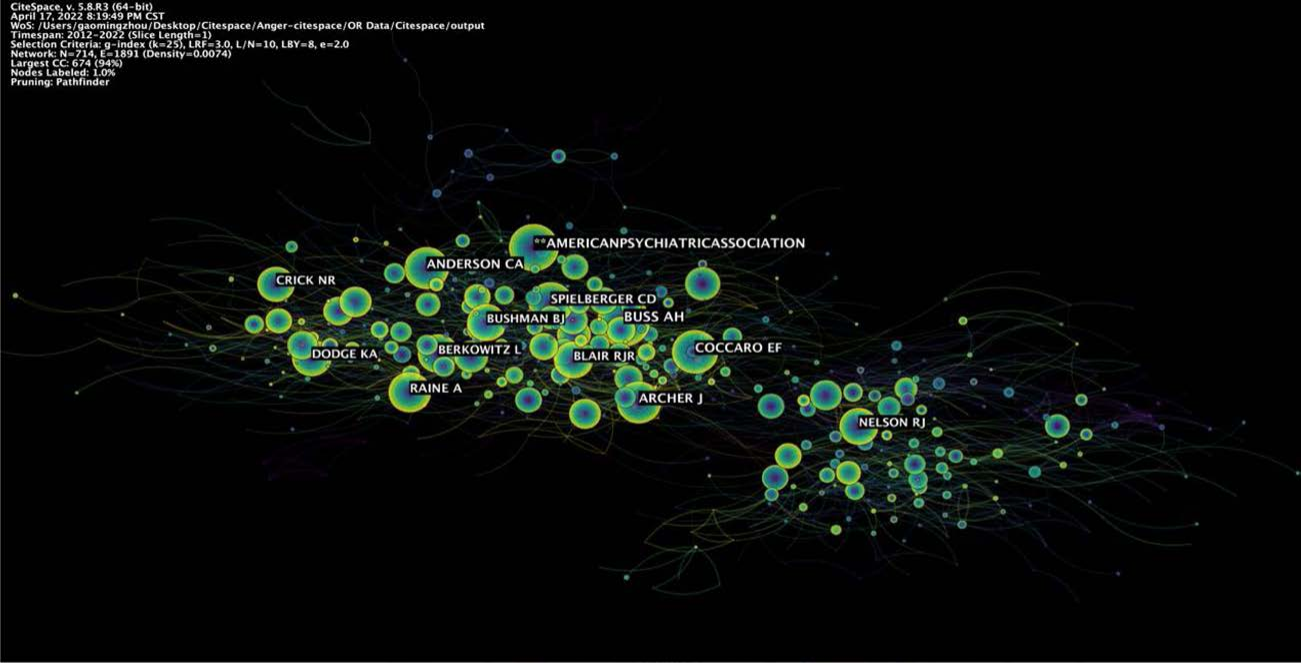
Map demonstrating co-authorship in anger and aggression research. The bigger the circle, the more original the articles the author published. The shorter and thicker the connection line, the closer the relationship between authors.

A total of 505 authors contributed to the literature on anger and aggression. The analysis showed that Emil F. Coccaro was the most prolific author who published 22 articles on anger and aggression. However, the authors lack cooperation, apart from only a large cooperative network (Fig. 5). The data showed that Jan K. Buitelaar (0.03), Robert L. Findling (0.02), and Jonathan Mill (0.02) have high centralities, which shows that they have a strong influence on each other work as well as research from other groups (Fig. 6, Table 3).

**Table 3:** Map of co-cited authors in anger and aggression research in the past 10 yr.

Rank	Author	Count	Co-Author	Citation
1	Emil F. Coccaro	23	Buss A.H.	339
2	David S. Chester	14	American Psychiatric Association	253
3	Robert L. Findling	12	Archer J	219
4	C. Nathan Dewall	12	Coccaro E.F.	218
5	Royce Lee	12	Anderson C.A.	211
6	Jan K. Buitelaar	10	Raine A.	194
7	Ute Habel	9	Spielberger C.D.	189
8	Katja Bertsch	9	Bushman B.J.	160
9	Brooke S.G. Molina	8	Dodge K.A.	159
10	L. Eugene Arnold	8	Berkowitz L.	157

Co-cited authors are 2 or more authors cited by another or more papers simultaneously and constitute a co-cited relationship. Of 713 co-cited authors, ten have been cited more than 157 times (Table 2). BUSS AH (339) is the most cited author.

### 3.4. Analysis of journals and cited journals

Our analysis showed that 3114 publications were published in 206 journals. Out of these journals, AGGRESSIVE BEHAVIOR published the most papers (60), followed by SCIENTIFIC REPORTS (53), and ANIMALS (31) (Table 4). AGGRESSIVE BEHAVIOR aims to publish research on mechanisms underlying or influencing behaviors generally regarded as aggressive and the physiological and/or behavioral consequences of being subject to such behaviors. The journal with the highest impact factors was NEUROPHARMACOLOGY, which considers papers in any area of neuroscience.

**Table 4:** The top 10 academic journals publishing studies on anger and aggression in the past 10 yr.

Rank	Publications	Journal	IF
1	60	AGGRESSIVE BEHAVIOR	JCR: Q2 2.917
2	53	SCIENTIFIC REPORTS	JCR: Q1 4.38
3	31	ANIMALS	JCR: Q1 2.752
4	11	INTERNATIONAL JOURNAL OF ENVIRONMENTAL RESEARCH AND PUBLIC HEALTH	JCR: Q1 3.39
5	8	NEUROPHARMACOLOGY	JCR: Q1 5.251
6	8	PLOS ONE	JCR: Q2 3.24
7	8	JOURNAL OF AFFECTIVE DISORDERS	JCR: Q1 4.839
8	8	JOURNAL OF CHILD AND ADOLESCENT PSYCHOPHARMACOLOGY	JCR: Q2 2.576
9	7	ANIMAL BEHAVIOUR	JCR: Q1 2.844
10	6	ETHOLOGY	JCR: Q2 1.897

**Figure 7.**
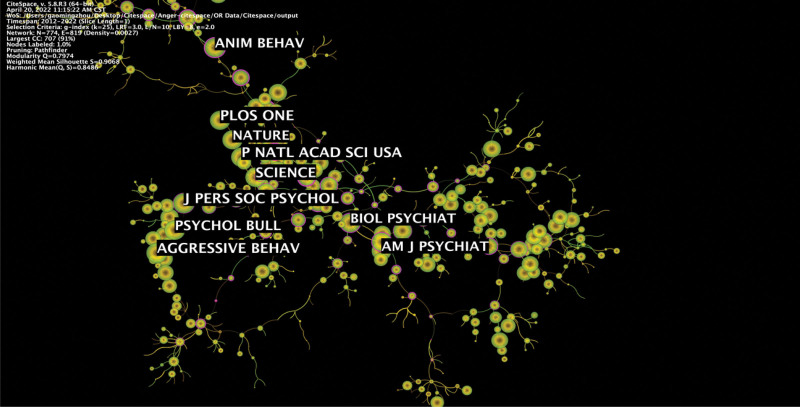
Map of journal co-citation on anger and aggression research.

As shown in Table 5 and Figure 7, the top 10 co-cited journals were PLOS ONE, J PERS SOC PSYCHOL, AGGRESSIVE BEHAV, P NATL ACAD SCI USA, SCIENCE, PSYCHOL BULL, ANIM BEHAV, AM J PSYCHIAT, BIOL PSYCHIAT and PLOS ONE. On the other hand, the top 10 journals in centrality were DEV PSYCHOBIOL, EUR J PHARMACOL, DRUG ALCOHOL DEPEN, AM J PRIMATOL, BEHAVIOUR, PSYCHONEUROENDOCRINO, TRENDS COGN SCI, ANIM BEHAV, J NEUROSCI, and DEV PSYCHOBIOL.

**Table 5:** Top 10 co-cited journals with the highest frequency and centrality of anger and aggression research in the past 10 yr.

Rank	Journal	Co-Citation	Journal	Centrality
1	PLOS ONE	962	DEV PSYCHOBIOL	0.11
2	J PERS SOC PSYCHOL	858	EUR J PHARMACOL	0.09
3	AGGRESSIVE BEHAV	826	DRUG ALCOHOL DEPEN	0.08
4	P NATL ACAD SCI USA	825	AM J PRIMATOL	0.08
5	SCIENCE	740	BEHAVIOUR	0.07
6	PSYCHOL BULL	709	PSYCHONEUROENDOCRINO	0.07
7	ANIM BEHAV	634	TRENDS COGN SCI	0.07
8	AM J PSYCHIAT	617	ANIM BEHAV	0.06
9	BIOL PSYCHIAT	606	J NEUROSCI	0.06
10	NATURE	596	DEV PSYCHOBIOL	0.11

### 3.5. Analysis of co-cited references

**Figure 8.**
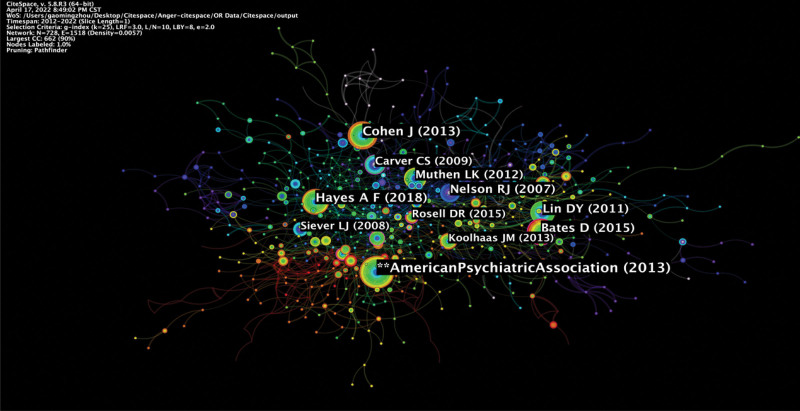
Map of reference co-citation related to anger and aggression studies.

The top ten cited references about frequency and centrality are shown in Table 6, Table 7, and Figure 8. According to the ranking of frequency and centrality in cited references, most were review papers, and a few were original research papers. The first ranked citation in terms of frequency was “diagnostic and statistical manual of mental disorders (5th ed.)” with a co-citation count of 93 (Fig. 8). Diagnostic and statistical manual of mental disorders is known as the “Bible” in the field of mental disorders.^[21]^ It is a guide manual for diagnosing mental disorders commonly used in the United States and other countries, including China. In diagnostic and statistical manuals of mental disorders, disorders related to anger or aggression have been included and frequently used to diagnose related diseases^[22,23]^ (Table 6).

**Table 6:** Top10 co-cited references on the highest frequency of anger and aggression research in the past 10 yr.

Rank	Counts	First author, Yr	Co-cited reference
1	93	American Psychiatric Association, 2013	Diagnostic and statistical manual of mental disorders (5th ed.)
2	58	Cohen J., 2013	Statistical power analysis for the behavioral sciences
3	48	Lin D.Y., 2011	Functional identification of an aggression locus in the mouse hypothalamus
4	48	Nelson R.J., 2007	Neural mechanisms of aggression
5	34	Carver C.S., 2009	Anger is an approach-related affect: Evidence and implications
6	33	Siever L.J., 2008	Neurobiology of aggression and violence
7	30	Rosell D.R., 2015	The neurobiology of aggression and violence
8	28	Koolhaas J.M., 2013	The resident-intruder paradigm: A standardized test for aggression, violence and social stress
9	23	Coccaro E.F., 2007	Amygdala and orbitofrontal reactivity to social threat in individuals with impulsive aggression
10	18	Blair R.J.R., 2016	The neurobiology of impulsive aggression

**Table 7:** Top 10 co-cited references for the highest centrality of anger and aggression research in the past 10 yr.

Rank	Centrality	First author, Yr	Cited reference
1	0.12	Coccaro E.F., 2011	Corticolimbic function in impulsive aggressive behavior
2	0.11	Rosell D.R., 2015	The neurobiology of aggression and violence
3	0.11	Duke A.A., 2013	Revisiting the serotonin-aggression relation in humans: A meta-analysis
4	0.1	Carver C.S., 2009	Anger is an approach-related affect: Evidence and implications
5	0.1	Cima M., 2013	The rewarding effect of aggression is reduced by nucleus accumbens dopamine receptor antagonism in mice
6	0.1	Couppis M.H., 2008	The rewarding effect of aggression is reduced by nucleus accumbens dopamine receptor antagonism in mice
7	0.1	Buades-Rotger M., 2016	Endogenous testosterone is associated with lower amygdala reactivity to angry faces and reduced aggressive behavior in healthy young women
8	0.09	Koolhaas J.M., 2013	The resident-intruder paradigm: A standardized test for aggression, violence and social stress
9	0.09	Blair R. J.R., 2016	The neurobiology of impulsive aggression
10	0.08	American Psychiatric Association, 2013	Diagnostic and statistical manual of mental disorders (5th ed.)

About the centrality of the cited references, the study ranked first was “Corticolimbic Function in Impulsive Aggressive Behavior.” This review discussed 3 neural systems involved in impulsive/reactive aggression: subcortical neural systems, decision-making circuits, social-emotional information processing circuits, and frontoparietal regions. The above neural systems are related to psychiatric disorders characterized by aggression^[24]^ (Table 7).

### 3.6. Analysis of keywords

#### 3.6.1. Keyword co-occurrence.

**Figure 9.**
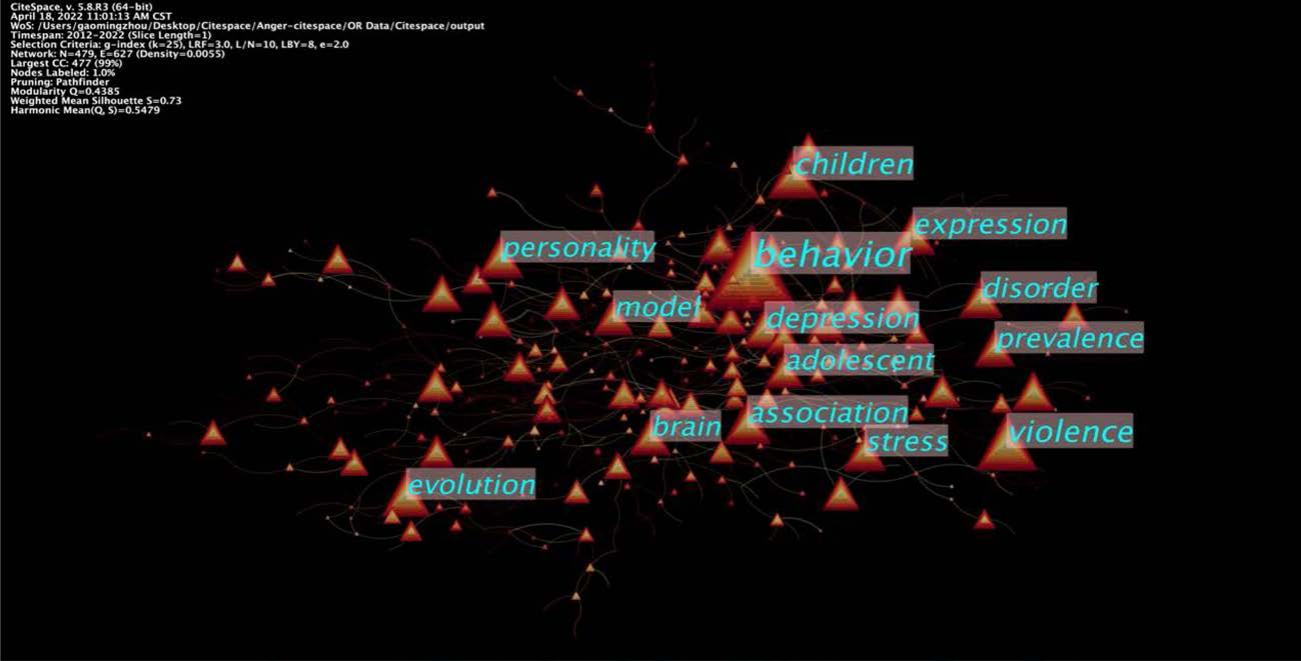
Keyword co-occurrence map of anger and aggression.

As shown in Table 8, an analysis of co-occurrence frequency revealed that behavior, violence, children, association, expression, depression, stress, evolution, personality, and adolescent were the main keywords. On the other hand, an analysis of co-occurrence centrality (Table 8, Fig. 9) demonstrated that the major keywords were anger, aggressive behavior, dominance, association, impulsivity, life history, brain, sex difference, physical aggression, and risk factor.

**Table 8:** Top 10 keywords on frequency and centrality in anger and aggression research in the past 10 yr.

Ranking	Counts	Keyword	Centrality	Keyword
1	778	Behavior	0.09	Anger
2	240	Violence	0.08	Aggressive behavior
3	179	Children	0.08	Dominance
4	155	Association	0.06	Association
5	144	Expression	0.06	Impulsivity
6	142	Depression	0.05	Life history
7	137	Stress	0.04	Brain
8	134	Evolution	0.04	Sex difference
9	134	Personality	0.04	Physical aggression
10	131	Adolescent	0.04	Risk factor

#### 3.6.2. Burst detection.

**Figure 10.**
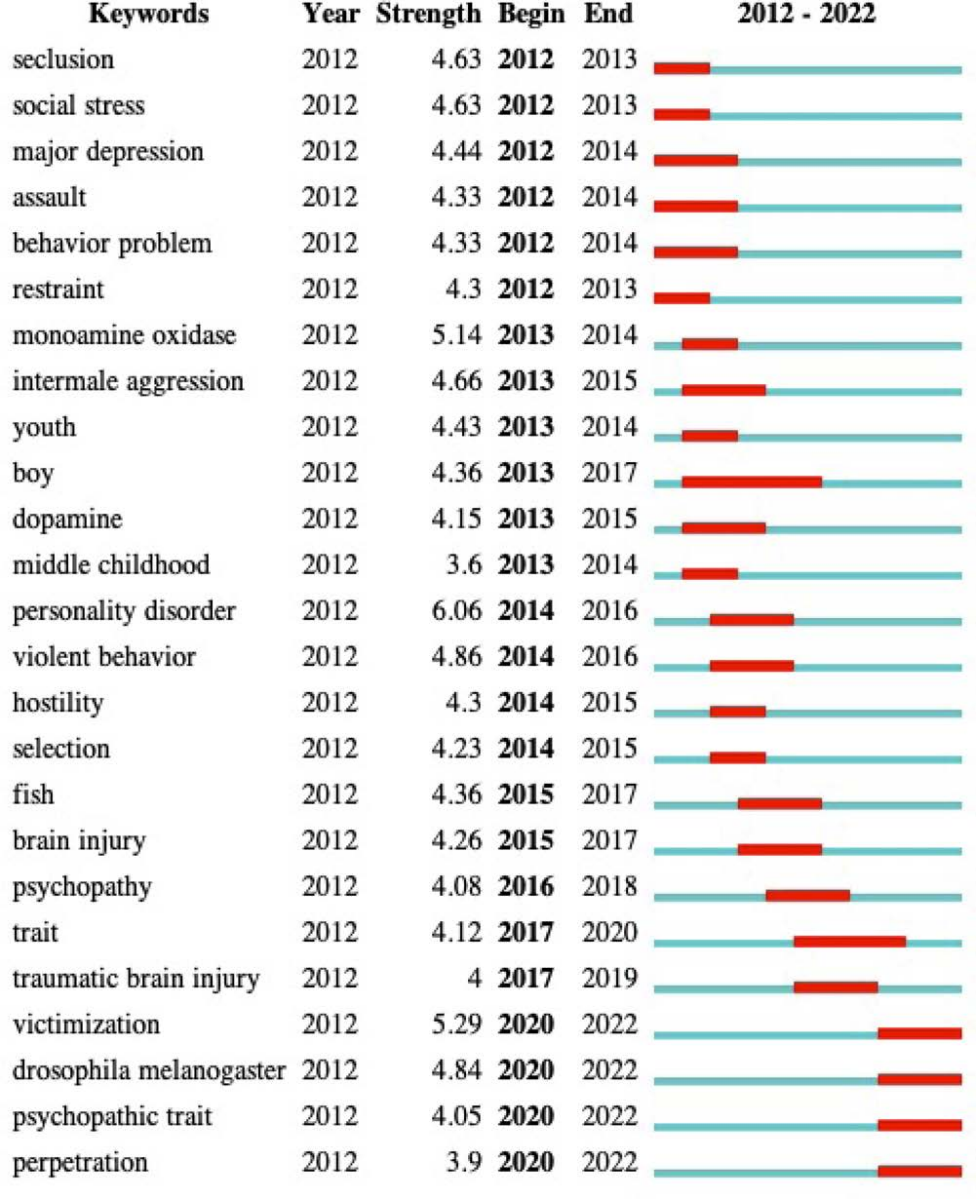
Top 25 keywords with the strongest citation bursts. The red bars demonstrate that some keywords were frequently cited, while the green bars show the keywords that were less frequently cited.

Keywords with citation bursts demonstrate that the keywords have higher citations in a given period, which can be regarded as emerging topics. The analysis of burst keywords showed that victimization, drosophila melanogaster, psychopathic trait, and perpetration are emerging trends of anger and aggression research (Fig. 10).

## 4. Discussion

### 4.1. General status

Anger is as harmful to people as depression and anxiety, which are associated with a higher risk of cardiovascular events but are often neglected.^[25]^ This study collected and analyzed 3114 articles from the WoSCC from April 17, 2002 to April 17, 2022. The data showed that although the number of publications steadily increased, there was a decline from 2019, suggesting that anger and aggression research is still attractive.

The 3114 articles emanated from 106 countries and 381 institutions. Among the contributing countries, the USA is the most productive country (1284 publications), with the highest centrality (0.26), suggesting its leading role in this research. Out of 381 institutions, Univ Penn ranked first in the number of publications and centrality, ranked 13th in the 2022 US. News World University report and the Thames Higher Education World University in 2022. The data also showed that 505 authors were involved in publishing literature on anger and aggression research. Emil F. Coccaro (Ohio State Univ in the USA) was the most prolific author. Jank Buitelaar (0.03), Robert L. Findling (0.02), and Jonathan Mill (0.02) have a strong influence on each other work as well as studies from other groups. The 4 are core authors in the field of anger and aggression research. Besides, research related to anger and aggression is often published on AGGRESSIVE BEHAVIOR, SCIENTIFIC REPORTS, and ANIMALS. In addition, analysis of co-cited journals revealed that publications in PLOS ONE and DEV PSYCHOBIOL had the greatest impact on the current research.

### 4.2. Knowledge base

In bibliometrics, the frontier in a field of research represents the current developmental state of a discipline, and the references in the frontier article constitute the intellectual base of the field. We identified the highest analyzed neural mechanisms of anger and aggression by analyzing the references. Anger and aggression involve very complex mechanisms and are related to decreased activation in the frontal brain regions and the dorsal anterior cingulate cortex and relatively fewer amygdala activation,^[26]^ especially the dorsolateral prefrontal cortex in the regulation of aggressive social behavior.^[27]^

### 4.3. Research hotspots and trends

#### 4.3.1. Research hotspots.

A hotspot is a scientific issue or topic discussed in a consortium that is intrinsically linked in a certain period, which high-frequency keywords could determine. In this study, we analyzed high-frequency keywords. We demonstrated that the top 10 keywords with co-occurrence frequency were behavior, violence, children, association, expression, depression, stress, evolution, personality, and adolescence. Besides, the ten keywords regarding co-occurrence centrality were anger, aggressive behavior, dominance, association, impulsivity, life history, brain, sex difference, physical aggression, and risk factors. The findings demonstrated that expression and behaviors of anger and aggression, physical aggression and risk factors, neural mechanism, personality, and adolescence had been researched hotspots in the past ten years.

To a certain extent, aggressive behavior reflects the level of anger. Anger and aggression research centers on expressing and controlling anger.^[28,29]^ In society, anger levels and expression significantly influence the development of generalized anxiety disorder and the level of anxiety.^[30]^ Uncontrolled anger is most commonly associated with personality disorders.^[28]^ Thus, anger is a common study term. Besides, the association between anger and the brain, such as amygdala volume^[31]^ and corpus callosum^[32]^ remains a hotspot. On the other hand, age was the most common risk factor affecting anger management in physicians.^[33]^ Besides, anger is regarded as risk factor in diseases, such as coronary artery disease^[34]^ and type 2 diabetes mellitus.^[35]^ Research data indicated that adolescents exhibit poor ability to control their anger and aggression, thus commonly studied.^[4]^

#### 4.3.2. Research trends.

Keywords with citation bursts indicate the keywords with higher citations in a given period, which can be regarded as highlighting emerging topics. Through analysis of burst keywords, victimization, drosophila melanogaster, psychopathic trait, and perpetration are emerging trends in anger and aggression research.

Victimization due to anger and aggression is one of the research directions in the future.^[36,37]^ In school, peer victimization is a significant public health problem associated with higher levels of teacher-rated aggressive behavior.^[38]^ In addition, previous studies reported that adolescents with asthma were subjected to more peer victimization and experienced more difficulties in anger expression.^[39]^ Besides, it was reported that anger and aggression lead to destructive results, such as violence and frequent interpersonal conflicts. Recent studies demonstrate that 41% of female drinkers perpetrated intimate partner violence towards their current partner.^[40]^ Besides, drosophila melanogaster, the model animal for genetics and neuroscience, might help develop new treatment options for clinical entities such as aggression and anxiety disorders.^[41]^ Also, drosophila melanogaster has been regarded as a new model organism for neurobiology of aggression.^[42,43]^ However, aggression of drosophila melanogaster is not related to human emotion, aggression led by anger emotion is still frequently studied by rats.^[44]^

## 5. Strengths and limitations

This is the first study to use CiteSpace to perform bibliometric analysis and visually display publications on anger and aggression research. However, our study still has some limitations. Only studies written in English were included. Thus, our results may be inapplicable to research published in other languages. However, the existing research results can already reflect the research trend in this field internationally.

## 6. Conclusion

Anger and aggression research has gained significant attention in the past ten years. Our analysis showed that the USA and Univ Penn are leading among countries and institutions. Emil F. Coccaro and Buss A.H. were core authors with significant output in this field. AGGRESSIVE BEHAVIOR was very active, while publications in PLOS ONE had the biggest influence on subsequent research. In addition, behaviors of anger and aggression, physical aggression and risk factors, neural mechanisms, personality, and adolescence have been the research hotspots in the past ten years. Victimization, drosophila melanogaster, psychopathic traits, and perpetration are emerging trends in anger and aggression research.

## Author contributions

**Conceptualization:** Xiaowen Sun, Xufeng Yu.

**Data curation:** Xufeng Yu.

**Formal analysis:** Xufeng Yu.

**Funding acquisition:** Kejian Li.

**Investigation:** Xufeng Yu, Kejian Li.

**Methodology:** Xiaowen Sun, Xufeng Yu.

**Project administration:** Xufeng Yu.

**Resources:** Xiaowen Sun, Kejian Li.

**Software:** Xiaowen Sun.

**Supervision:** Kejian Li.

**Visualization:** Xiaowen Sun.

**Writing – original draft:** Xiaowen Sun.

**Writing – review & editing:** Kejian Li, Xiaowen Sun.
